# Bias in logistic regression due to imperfect diagnostic test results and practical correction approaches

**DOI:** 10.1186/s12936-015-0966-y

**Published:** 2015-11-04

**Authors:** Denis Valle, Joanna M. Tucker Lima, Justin Millar, Punam Amratia, Ubydul Haque

**Affiliations:** School of Forest Resources and Conservation, University of Florida, Gainesville, USA; Emerging Pathogens Institute, University of Florida, Gainesville, USA; Geography Department, University of Florida, Gainesville, USA

**Keywords:** Imperfect detection, Misclassification, Sensitivity, Specificity, Risk factor, Logistic regression, Bias, Diagnostic test

## Abstract

**Background:**

Logistic regression is a statistical model widely used in cross-sectional and cohort studies to identify and quantify the effects of potential disease risk factors. However, the impact of imperfect tests on adjusted odds ratios (and thus on the identification of risk factors) is under-appreciated. The purpose of this article is to draw attention to the problem associated with modelling imperfect diagnostic tests, and propose simple Bayesian models to adequately address this issue.

**Methods:**

A systematic literature review was conducted to determine the proportion of malaria studies that appropriately accounted for false-negatives/false-positives in a logistic regression setting. Inference from the standard logistic regression was also compared with that from three proposed Bayesian models using simulations and malaria data from the western Brazilian Amazon.

**Results:**

A systematic literature review suggests that malaria epidemiologists are largely unaware of the problem of using logistic regression to model imperfect diagnostic test results. Simulation results reveal that statistical inference can be substantially improved when using the proposed Bayesian models versus the standard logistic regression. Finally, analysis of original malaria data with one of the proposed Bayesian models reveals that microscopy sensitivity is strongly influenced by how long people have lived in the study region, and an important risk factor (i.e., participation in forest extractivism) is identified that would have been missed by standard logistic regression.

**Conclusion:**

Given the numerous diagnostic methods employed by malaria researchers and the ubiquitous use of logistic regression to model the results of these diagnostic tests, this paper provides critical guidelines to improve data analysis practice in the presence of misclassification error. Easy-to-use code that can be readily adapted to WinBUGS is provided, enabling straightforward implementation of the proposed Bayesian models.

**Electronic supplementary material:**

The online version of this article (doi:10.1186/s12936-015-0966-y) contains supplementary material, which is available to authorized users.

## Background

Epidemiologists use logistic regression to identify risk factors (or protective factors) based on binary outcomes from diagnostic tests. As a consequence, this statistical model is used ubiquitously in studies conducted around the world, encompassing a wide range of diseases. One issue with this tool, however, is that it fails to account for imperfect diagnostic test results (i.e., misclassification errors). In other words, depending on the diagnostic method employed, a negative test might be incorrectly interpreted as lack of infection (i.e., false-negative) [1–3] and/or a positive test result might be incorrectly interpreted as infection presence (i.e., false-positive) [3–8]. This is particularly relevant for malaria given the numerous diagnostic techniques that are commonly employed [e.g., rapid diagnostic tests (RDTs), fever, anaemia, microscopy, and polymerase chain reaction (PCR)].

Imperfect detection has important implications. For instance, the determination of infection prevalence (i.e., the proportion of infected individuals) will be biased if detection errors are ignored [9–11]. However, it is typically under-appreciated that errors in detection may also influence the identification of risk factors and estimates of their effect. An important study by Neuhaus [12] demonstrated that as long as covariates do not influence sensitivity and/or specificity (e.g., non-differential outcome misclassification), then imperfect detection is expected to result in adjusted odds ratios that are artificially closer to zero and underestimation of uncertainty in parameter estimates (see also [13]). However, when sensitivity and specificity are influenced by covariates, the direction of the bias in parameter estimates is difficult to predict [12, 14].

Several methods have been proposed in the literature to adjust for misclassification of outcomes, including an expectation–maximization (EM) algorithm [15], the explicit acknowledgement of misclassification in the specification of the likelihood, enabling users to fit the model using SAS code [16], probabilistic sensitivity analysis [17] and Bayesian approaches [18]. Unfortunately, these methods have not been widely adopted by the malaria epidemiology community, likely because these problems are rarely acknowledged outside biostatistics and statistically inclined epidemiologists. Lack of awareness is particularly problematic because several of the proposed modelling approaches that address this problem work best if an ‘internal validation sample’ is collected alongside the main data.

This article begins with a brief literature review to demonstrate how malaria epidemiologists are generally unaware of the problem associated with, and the proposed methods to deal with, misclassification error. Then, different types of auxiliary data and the associated statistical models that can be used to appropriately address this problem are described and straightforward code is provided to readily implement these models. Finally, performance of these models is illustrated using simulations and a case study on malaria in a rural settlement of the western Brazilian Amazon.

## Methods

### Systematic literature review

To provide support for the claim that malaria epidemiologists generally do not modify their logistic regressions to account for imperfect diagnostic test outcomes, a targeted literature review was conducted. PubMed was searched using different combinations of the search terms ‘malaria’, ‘logistic’, ‘models’, ‘regression’, ‘diagnosis’, and ‘diagnostic’. The search was restricted to studies published between January 2005 and April 2015. Of the 209 search results, 173 articles were excluded because they included authors from this article, were unrelated to malaria, malarial status was either unreported or not the outcome variable in the logistic regression, and/or they relied solely on microscopy. Studies that relied only on microscopy were excluded because this diagnostic method is considered the gold standard in much of the world, with the important exception of locations with relatively low transmission (e.g., Latin America), where PCR is typically considered to be the gold standard method. Detailed information regarding the literature review (e.g., list of articles with the associated reasons for exclusion) is available upon request.

### Statistical models and auxiliary data to address misclassification error

To avoid the problem associated with imperfect detection when using logistic regression, one obvious solution is to use a highly sensitive and specific diagnostic test (e.g., the gold standard method) to determine disease status for all individuals. Unfortunately, this is often unfeasible and/or not scalable because of cost or other method requirements (e.g., electricity, laboratory equipment, expertise availability, or time required). Alternatively, statistical methods that specifically address the problem of imperfect detection (i.e., misclassification) can be adopted. Unfortunately, these statistical models contain parameters that cannot be estimated from data collected in regular cross-sectional surveys or cohort studies based on a single diagnostic test. Therefore, these statistical methods are described in detail along with the additional data that are required to fit them.

For all models, JAGS code is provided for readers interested in implementing and potentially modifying these models (see Additional Files 1, 2, 3, and 4 for details). Readers should have no problem adapting the same code to WinBUGS/OpenBUGS, if desired. The benefit of using Bayesian models is that they can be readily extended to account for additional complexities (e.g., random effects to account for sampling design). As a result, the code provided here is useful not only for users interested in this paper’s Bayesian models but also as a stepping stone for more advanced models.

### Bayesian model 1

One option is to use results from an external study on the sensitivity and specificity of the diagnostic method employed. Say that this external study employed the same diagnostic method, together with the gold standard method, and reported the estimated sensitivity $\widehat{SN}$ and specificity $\widehat{SP}$. This information can be used to properly account for imperfect detection. More specifically, Bayesian model 1 assumes that

$$I_{i} \sim Bernoulli\left( {\frac{{{ \exp }\left( {\beta_{0} + \beta_{1} x_{i1} + \beta_{2} x_{i2} + \cdots } \right)}}{{1 + { \exp }\left( {\beta_{0} + \beta_{1} x_{i1} + \beta_{2} x_{i2} + \cdots } \right)}}} \right)$$

where $I_{i}$ is the infection status of the ith individual, $\beta_{0} ,\beta_{1} ,\beta_{2} , \ldots$ are regression parameters, and ${\text{x}}_{{{\text{i}}1}} ,x_{i2} , \ldots$ are covariates. It further assumes that:

$$D_{i} \sim Bernoulli(\widehat{SN}) \quad {\text{if}}\;\;I_{i} = 1$$

$$D_{i} \sim Bernoulli\left( {1 - \widehat{SP}} \right) \quad {\text{if}}\;I_{i} = 0$$

where $D_{i}$ is the regular diagnostic test result for the ith individual. Finally, different priors can be assigned for the disease regression parameters. A fairly standard uninformative prior is adopted for these parameters, given by:

$$\beta_{j} \sim N(0,10).$$

One problem with this approach, however, is that it assumes that these diagnostic test parameters are exactly equal to their estimates $\widehat{SN}$ and $\widehat{SP}$. A better approach would account for uncertainty around these estimates of sensitivity and specificity, as described in Bayesian model 2.

### Bayesian model 2

This model is very similar to Bayesian model 1, except that it employs informative priors for sensitivity SN and specificity SP. One way to create these priors is to use the following information from the external study:

- $N_{ + }$ number of infected individuals, as assessed using the gold standard method;
- $T_{ + }$ number of individuals detected to be infected by the regular diagnostic method among all $N_{ + }$ individuals;
- $N_{ - }$ number of healthy individuals, as assessed using the gold standard method; and
- $T_{ - }$ number of individuals not detected to be infected by the regular diagnostic method among all $N_{ - }$ individuals.

Following the ideas in [19, 20], these ‘data’ can be used to devise informative priors of the form:

$$SN\sim Beta\left( {T_{ + } + 1,N_{ + } - T_{ + } + 1} \right)$$

$$SP\sim Beta\left( {T_{ - } + 1,N_{ - } - T_{ - } + 1} \right).$$

There are other ways of creating informative priors for *SN* and *SP* that do not rely on these four numbers (i.e., $T_{ - } ,T_{ + } ,N_{ - } ,N_{ + }$) (e.g., based on estimates of *SN* and *SP* with confidence intervals from a meta-analysis) but the method proposed above is likely to be broadly applicable given the abundance of studies that report these four numbers.

Two potential problems arise when using external data to estimate *SN* and *SP*. First, results from the external study are assumed to aptly apply to the study in question (i.e., ‘transportability’ assumption), which may not necessarily be the case if diagnostic procedures and storage conditions of diagnostic tests are substantially different. Second, the performance of the diagnostic test may depend on covariates (i.e., differential misclassification) [16]. For instance, microscopy performance for malaria strongly depends on parasite density [21]. If age is an important determinant of parasite density in malaria (i.e., older individuals are more likely to display lower parasitaemia), then microscopy sensitivity might be higher for younger children than for older children or adults. Another example refers to diagnostic methods that rely on the detection of antibodies. For these methods, sensitivity might be lower for people with compromised immune systems (e.g., malnourished children). In these cases, adopting a single value of *SN* and *SP* in Bayesian model 1 or 2 might be overly simplistic and may lead to even greater biases in parameter estimates. Bayesian model 3 solves these two problems associated with using external data.

### Bayesian model 3

Instead of relying on external sources of information, another alternative is to collect additional information on the study participants themselves (also known as an internal validation sample [16]). More specifically, due to its higher cost, one might choose to diagnose only a small sub-set of individuals using the gold standard method. This sample enables the estimation of *SN* and *SP* of the regular diagnostic test (and potentially reveals how these test performance characteristics are impacted by covariates) without requiring the ‘transportability’ assumption associated with using external data.

In Bayesian model 3, the gold standard method is assumed to be employed concurrently with the regular diagnostic method for a randomly chosen sub-set of individuals. Its structure closely follows that of Bayesian models 1 and 2, except that now sensitivity and specificity are allowed to vary according to covariates:

$$D_{i} \sim Bernoulli\left( {SN_{i} = \frac{{{ \exp }\left( {\alpha_{0} + \alpha_{1} x_{i1} + \alpha_{2} x_{i2} + \cdots } \right)}}{{1 + { \exp }\left( {\alpha_{0} + \alpha_{1} x_{i1} + \alpha_{2} x_{i2} + \cdots } \right)}}} \right)\;{\text{if}}\;I_{i} = 1$$

$$D_{i} \sim Bernoulli\left( {1 - SP_{i} = \frac{1}{{1 + { \exp }\left( {\omega_{0} + \omega_{1} x_{i1} + \omega_{2} x_{i2} + \cdots } \right)}}} \right)\;{\text{if}}\;I_{i} = 0$$

where additional regression parameters ($\alpha_{0} ,\alpha_{1} ,\alpha_{2} , \ldots$ and $\omega_{0} ,\omega_{1} ,\omega_{2} , \ldots$) determine how sensitivity and specificity, respectively, vary from individual to individual as a function of the observed covariates. Notice that the covariates in these sensitivity and specificity sub-models do not need to be the same as those used to model infection status $I_{i}$. Also notice that it is only feasible to estimate all these regression parameters because of the assumption that infection status $I_{i}$ is known for a sub-set of individuals tested with the gold standard method. More specifically, it is assumed that $I_{i} = G_{i}$ for these individuals, where $G_{i}$ is the result from the gold standard method. A summary of the different types of data discussed above and the corresponding statistical models is provided in Table 1.

**Table 1:** Summary of the proposed statistical models, their assumptions regarding the diagnostic method, and the additional data required to fit these models

Model	Additional data requirement	Assumptions related to detection
Standard logistic regression	None	Perfect detection (i.e., sensitivity and specificity equal to 100 %)
Bayesian model 1	Estimate of sensitivity $\widehat{SN}$ and specificity $\widehat{SP}$ based on external study	Sensitivity and specificity are perfectly known constants, equal to the estimates from external study
Bayesian model 2	Data on sensitivity and specificity (i.e., $N_{ + } ,T_{ + } ,N_{ - } ,T_{ - }$) from external study	Sensitivity and specificity are constants and external study provides reasonable prior information on sensitivity and specificity for the target study
Bayesian model 3	Subset of individuals diagnosed with the regular and the gold standard method	Sensitivity and specificity can vary as a function of covariates. This model does not rely on data from external study (i.e., does not rely on transportability assumption)

### Simulations

The effectiveness of the proposed Bayesian models in estimating the regression parameters was assessed using simulations. One hundred datasets were created for each combination of sensitivity (*SN* = 0.6 or *SN* = 0.9) and specificity (*SP* = 0.9 or *SP* = 0.98). Sensitivity and specificity values were chosen to encompass a wide spectrum of performance characteristics of diagnostic methods. Furthermore, it is assumed that sensitivity and specificity do not change as a function of covariates. Each dataset consisted of diagnostic test results for 2000 individuals, with four covariates standardized to have mean zero and standard deviation of one. In these simulations, infection prevalence when covariates were zero (i.e., $\frac{{\exp \left( {\beta_{0} } \right)}}{{1 + \exp \left( {\beta_{0} } \right)}}$) was randomly chosen to vary between 0.2 and 0.6 and slope parameters were randomly drawn from a uniform distribution between −2 and 2.

For each simulated dataset, the true slope parameters were estimated by fitting a standard logistic regression (‘Std.Log.’) and the Bayesian models described above. For the methods that relied on external study results, it was assumed that $N_{ - } = N_{ + } = 100$ and that $T_{ + } \sim Binomial\left( {N_{ + } ,SN} \right)$ and $T_{ - } \sim Binomial\left( {N_{ - } ,SP} \right)$. Therefore, the assumption for Bayesian model 1 (‘Bayes 1’) was that sensitivity and specificity were equal to $\widehat{SN} = \frac{{T_{ + } }}{{N_{ + } }}$ and $\widehat{SP} = \frac{{T_{ - } }}{{N_{ - } }}$. For Bayesian model 2 (‘Bayes 2’), the set of numbers $\left\{ {T_{ + } ,T_{ - } ,N_{ + } ,N_{ - } } \right\}$ was used to create informative priors for sensitivity and specificity. Finally, Bayesian model 3 (‘Bayes 3’), assumed that results from the gold standard diagnostic method were available for an internal validation sample consisting of a randomly chosen sample of 200 individuals (10 % of the total number of individuals).

Two criteria were used to compare the performance of these methods. The first criterion assessed how often these methods captured the true parameter values within their 95 % confidence intervals (CI). Thus, this criterion consisted of the 95 % CI coverage for dataset d and method m, given by $C_{d,m} = \frac{{\mathop \sum \nolimits_{j = 1}^{4} I\left( {\hat{\beta }_{{{\text{j}},{\text{d}},{\text{m}}}}^{\text{lo}} \; < \beta_{j,d} < \hat{\beta }_{{{\text{j}},{\text{d}},{\text{m}}}}^{\text{hi}} } \right)}}{4}$. In this equation, $\beta_{j,d}$ is the jth true parameter value for simulated data d, and $\hat{\beta }_{{{\text{j}},{\text{d}},{\text{m}}}}^{\text{lo}}$ and $\hat{\beta }_{{{\text{j}},{\text{d}},{\text{m}}}}^{\text{hi}}$ are the jth estimated lower and upper bounds of the 95 % CI. The function I() is the indicator function, which takes on the value of one if the condition inside the parentheses is true and zero otherwise. Given that statistical significance of parameters is typically judged based on these CIs, it is critical that these intervals retain their nominal coverage. Thus, $C_{d,m}$ values close to 0.95 indicate better models.

One problem with the 95 % CI coverage criterion, however, is that a model might have good coverage as a result of exceedingly wide intervals, a result that is undesirable. Thus, the second criterion consisted in a summary measure that combines both bias and variance, given by the mean-squared errors (MSE). This statistic was calculated for dataset d and method m as $MSE_{d,m} = \frac{{\mathop \sum \nolimits_{j = 1}^{4} E\left[ {\left( {\beta_{j,d} - \hat{\beta }_{j,d,m} } \right)^{2} } \right]}}{4}$, where $\hat{\beta }_{j,d,m}$ and $\beta_{j,d}$ are the jth slope estimate and true parameter, respectively. Smaller values of $MSE_{d,m}$ indicate better model performance.

### Case study

Case study data came from a rural settlement area in the western Brazilian Amazon state of Acre, in a location called Ramal Granada. These data were collected in four cross-sectional surveys between 2004 and 2006, encompassing 465 individuals. Individuals were tested for malaria using both microscopy and PCR, regardless of symptoms. Additional details regarding this dataset can be found in [22, 23].

Microscopy test results were analyzed first using a standard logistic regression model, where the potential risk factors were age, time living in the study region (‘Time’), gender, participation on forest extractivism (‘Extract’), and hunting or fishing (‘Hunt/Fish’). Taking advantage of the concurrent microscopy and PCR results, the outcomes from this standard logistic regression model were then contrasted with that of Bayesian model 3.

Microscopy sensitivity is known to be strongly influenced by parasitaemia. Furthermore, it has been suggested that people in the Amazon region can develop partial clinical immunity (probably associated with lower parasitaemia) based on past cumulative exposure to low intensity malaria transmission [23–25]. Because rural settlers often come from non-malarious regions, time living in the region might be a better proxy for past exposure than age [23]. For these reasons, microscopy sensitivity was modelled as a function of age and time living in the region.

## Results

### Systematic literature review

Of the 36 studies that satisfied the criteria, 70 % did not acknowledge imperfect detection in malaria outcome. The only articles that accounted for imperfect detection were those exclusively focused on the performance of diagnostic tests [26–28]. No instances were found where imperfect detection was specifically incorporated into a logistic regression framework, despite the existence of methods to correct this problem within this modelling framework. These results suggest that malaria epidemiologists are generally unaware of the strong impact that imperfect detection can have on parameter estimates from logistic regression.

### Simulations

**Fig. 1:**
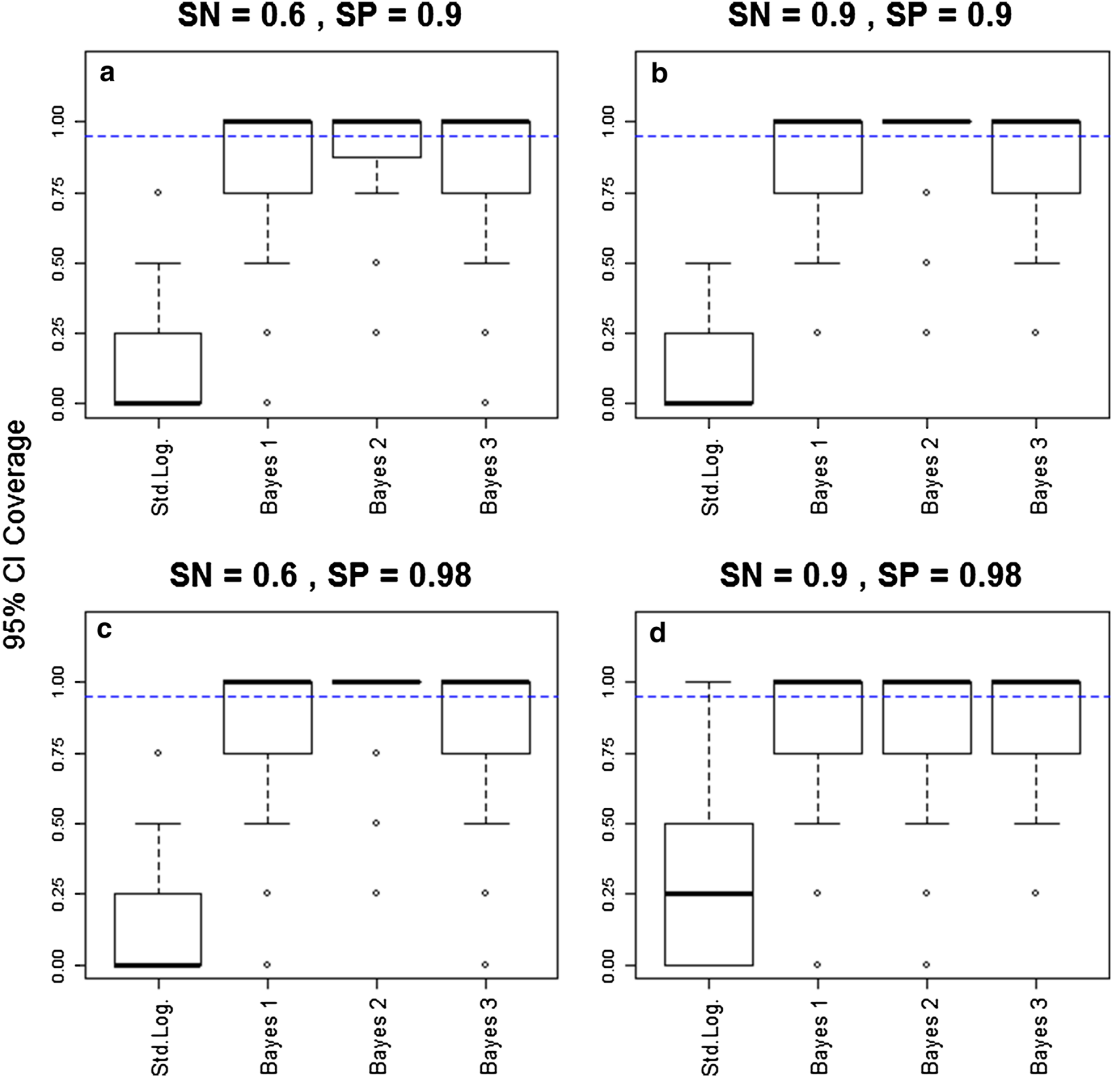
The proposed Bayesian models have a much better 95 % CI coverage than the standard logistic regression model. 95 % confidence/credible interval (CI) coverage for four different methods are shown for different scenarios of sensitivity (*SN*) and specificity (*SP*) [*SN* = 0.6 and *SP* = 0.9 (*upper left panel*); *SN* = 0.9 and *SP* = 0.9 (*upper right panel*); *SN* = 0.6 and *SP* = 0.98 (*lower left panel*); *SN* = 0.9 and *SP* = 0.98 (*lower right panel*)]. These results are based on 100 simulated datasets, with 2000 individuals in each dataset. Results closer to 0.95 (*blue horizontal dashed lines*) indicate better performance

Differences between the standard logistic regression and the proposed Bayesian models were striking regarding their 95 % credible interval (CI) coverage. The standard logistic regression had consistently lower than expected 95 % CI coverage, frequently missing the true parameter estimates (Fig. 1). For example, in the most optimistic scenario regarding the performance of the diagnostic method (scenario in which sensitivity and specificity were set to 0.9 and 0.98, respectively) only 6 % of the standard logistic regressions returned CIs that always contained the true parameter. On the other hand, the Bayesian models performed much better, frequently producing CIs that always contained the true parameters.

**Fig. 2:**
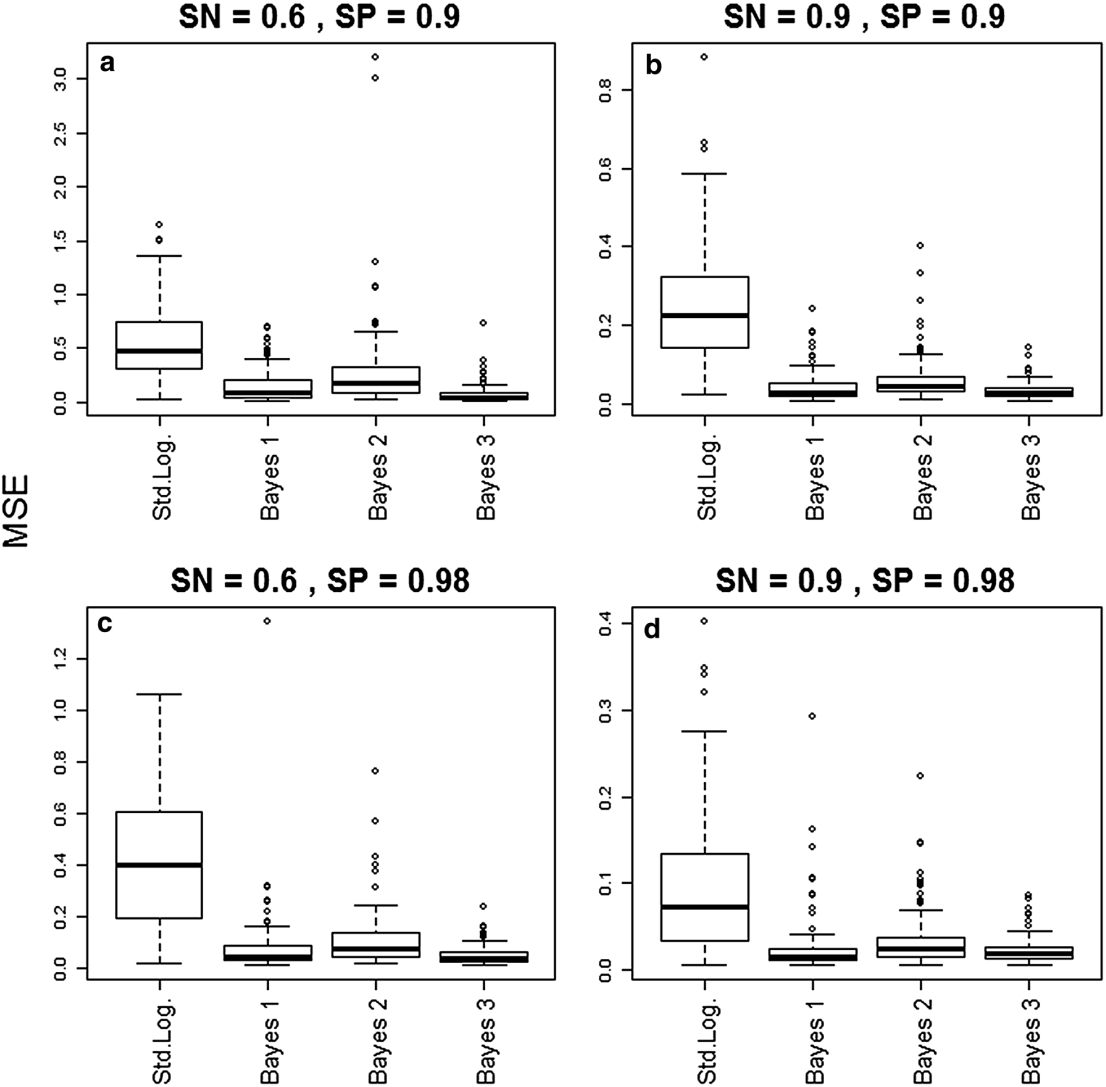
The Bayesian models outperformed the standard logistic regression model based on the MSE criterion. Mean squared error (MSE) for four different methods are shown for different scenarios of sensitivity (*SN*) and specificity (*SP*) [*SN* = 0.6 and *SP* = 0.9 (*upper left panel*); *SN* = 0.9 and *SP* = 0.9 (*upper right panel*); *SN* = 0.6 and *SP* = 0.98 (*lower left panel*); *SN* = 0.9 and *SP* = 0.98 (*lower right panel*)]. These results are based on 100 simulated datasets, with 2000 individuals in each dataset. *Smaller values* indicate better performance

Results also suggest that the improved 95 % CI coverage from the Bayesian models did not come at the expense of overly wide intervals. Indeed, these models greatly improved estimation of the true regression parameters under the MSE criterion compared to the standard logistic regression model (Fig. 2). The Bayesian models outperformed (i.e., had a smaller MSE) the standard logistic regression model in >78 % of the simulations. Finally, simulation results also revealed that diagnostic methods with low sensitivity and/or low specificity generally resulted in much higher MSE (notice the y-axis scale in Fig. 2), highlighting how imperfect detection can substantially hinder the ability to estimate the true regression parameters, regardless of the method employed to estimate parameters.

### Case study

**Fig. 3:**
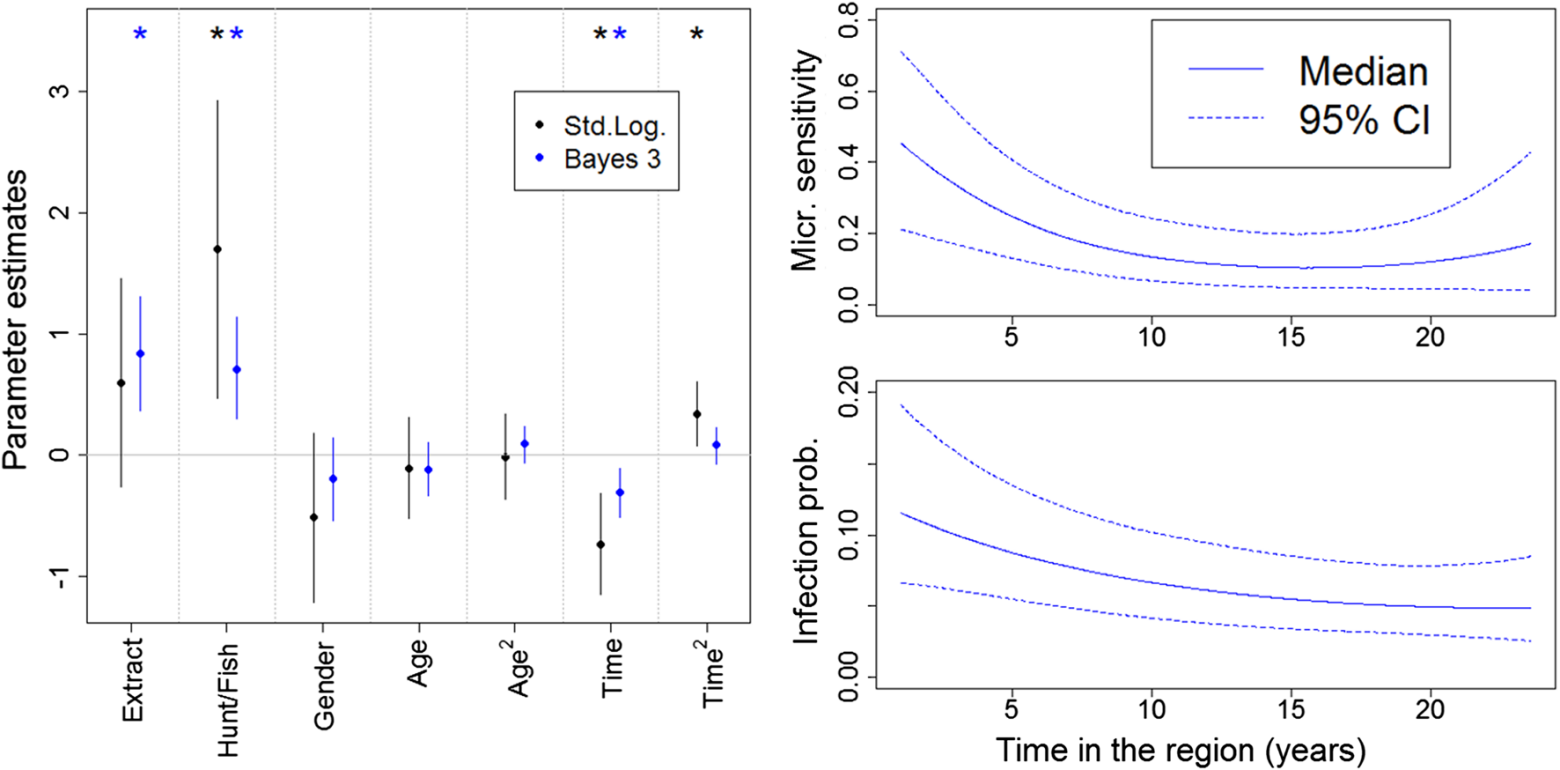
Results from the standard logistic regression (*black*) and Bayesian model 3 (*blue*). *Left panel* shows that inference regarding disease risk factors can be substantially different when using the standard logistic regression and Bayesian model 3. *Stars* indicate 95 % confidence/credible intervals that did not include zero. The ‘Time’ covariate refers to time of residence in the region. *Right panels* show that both microscopy sensitivity and infection probability decrease as a function of time living in this region

Findings reveal that the standard logistic regression results might fail to detect important risk factors (e.g., participation in forest extractivism ‘Extract’), might over-estimate some effect sizes (e.g., participation in hunting/fishing ‘Hunt/Fish’), or might incorrectly detect a significant quadratic relationship (e.g., ‘Time^2^’) (left panel in Fig. 3). The Bayesian model also suggests that settlers living for a longer period of time in the region tended to have lower parasitaemia, leading to a statistically significant lower microscopy sensitivity, as well as statistically significant lower probability of infection (right panels in Fig. 3). On the other hand, age was neither a significant covariate for sensitivity nor for probability of infection.

## Discussion

A review of the literature shows that malaria epidemiologists seldom modify their logistic regression to accommodate for imperfect diagnostic test results. Yet, the simulations and case study illustrate the pitfalls of this approach. To address this problem, three Bayesian models are proposed that, under different assumptions regarding data availability, appropriately accounted for sensitivity and specificity of the diagnostic method and demonstrated how these methods significantly improve inference on disease risk factors. Given the widespread use of logistic regression in epidemiological studies across different geographical regions and diseases and the fact that imperfect detection methods are not restricted to malaria, this article can help improve current data collection and data analysis practice in epidemiology. For instance, awareness of how imperfect detection can bias modelling results is critical during the planning phase of data collection to ensure that the appropriate internal validation dataset is collected if one intends to use Bayesian model 3.

Two of the proposed Bayesian models (‘Bayes 1’ and ‘Bayes 2’) rely heavily on external information regarding the diagnostic method (i.e., external validation data). As a result, if this information is unreliable, then these methods might perform worse than the simulations suggest. Furthermore, a key assumption in both of these models is that sensitivity and specificity do not depend on covariates (i.e., non-differential classification). This assumption may or may not be justifiable. Thus, a third model (‘Bayes 3’) was created which relaxes this assumption and relies on a sub-sample of the individuals being tested with both the regular diagnostic and gold standard methods (i.e., internal validation sample). For this latter model, one has to be careful regarding how the sub-sample is selected; if this sample is not broadly comparable to the overall set of individuals in the study (e.g., not a random sub-sample), biases might be introduced in parameter estimates [e.g., 29]. These three models are likely to be particularly useful for researchers interested in combining abundant data from cheaper diagnostic methods (e.g., data from routine epidemiological surveillance) with limited research data collected using the gold standard method [22, 30].

An important question refers to how to determine the size of the internal validation sample. To address this, it is important to realize that Bayesian model 3 encompasses three regressions: one for the probability of being diseased, another to model sensitivity and the third to model specificity. The sensitivity regression relies on those individuals diagnosed to be positive by the gold standard method while the specificity regression relies on those with a negative diagnosis using the gold standard method. As a result, if prevalence is low, then the sensitivity regression will have very few observations and therefore trying to determine the role of several covariates on sensitivity is likely to result in an overfitted model. Similarly, if prevalence is high, the specificity regression will have very few observations and care should be taken not to overfit the model. Ultimately, the necessary size of the internal validation sample will depend on overall disease prevalence (as assessed by the gold standard method) and the number of covariates that one wants to evaluate when modelling sensitivity and specificity. Finally, an important limitation of Bayesian model 3 is the assumption that the gold standard method performs perfectly (i.e., sensitivity and specificity equal to 1), which is clearly overly optimistic [31, 32]. Developing straightforward models that avoid the assumption of a perfect gold standard method represents an important area of future research.

Possible extensions of the model include allowing for correlated sensitivity and specificity or allowing for misclassification in response *and* exposure variables, as in [33, 34]. Furthermore, although this paper focused on the standard logistic regression, imperfect detection impacts other types of models as well, such as survival models [35] and Poisson regression models [20]. Finally, the benefits of using these models apply specifically to cross-sectional and cohort studies but not to case–control studies. In case–control studies, disease status is no longer random (i.e., it is fixed by design) and thus additional assumptions might be needed for the methods presented here to be applicable [16].

## Conclusions

The standard logistic regression model has been an invaluable tool for epidemiologists for decades. Unfortunately, imperfect diagnostic test results are ubiquitous in the field and may lead to considerable bias in regression parameter estimates. Given the numerous diagnostic methods employed by malaria researchers and the ubiquitous use of logistic regression to model the results of these diagnostic methods, this paper provides critical guidelines to improve data analysis practice in the presence of misclassification error. Easy-to-use code is provided that can be readily adapted to WinBUGS and enables straightforward implementation of the proposed Bayesian models. The time is ripe to improve upon the standard logistic regression and better address the challenge of modelling imperfect diagnostic test results.

## Additional files

10.1186/s12936-015-0966-y Code for the Bayesian models used in the main manuscript.
10.1186/s12936-015-0966-y Simulated data used to illustrate Bayesian model 1.
10.1186/s12936-015-0966-y Simulated data used to illustrate Bayesian model 2.
10.1186/s12936-015-0966-y Simulated data used to illustrate Bayesian model 3.

## References

[1] Barbosa S, Gozze AB, Lima NF, Batista CL, Bastos MDS, Nicolete VC (2014). Epidemiology of disappearing *Plasmodium vivax* malaria: a case study in rural Amazonia. PLOS Negl Trop Dis.

[2] Acosta POA, Granja F, Meneses CA, Nascimento IAS, Sousa DD, Lima Junior WP (2014). False-negative dengue cases in Roraima, Brazil: an approach regarding the high number of negative results by NS1 AG kits. Rev Inst Med Trop Sao Paulo.

[3] Weigle KA, Labrada LA, Lozano C, Santrich C, Barker DC (2002). PCR-based diagnosis of acute and chronic cutaneous leishmaniasis caused by Leishmania (Viannia). J Clin Microbiol.

[4] Baiden F, Webster J, Tivura M, Delimini R, Berko Y, Amenga-Etego S (2012). Accuracy of rapid tests for malaria and treatment outcomes for malaria and non-malaria cases among under-five children in rural Ghana. PLoS One.

[5] Peeling RW, Artsob H, Pelegrino JL, Buchy P, Cardosa MJ, Devi S (2010). Evaluation of diagnostic tests: dengue. Nat Rev Microbiol.

[6] Amato Neto V, Amato VS, Tuon FF, Gakiya E, de Marchi CR, de Souza RM (2009). False-positive results of a rapid K39-based strip test and Chagas disease. Int J Infect Dis..

[7] Sundar S, Reed SG, Singh VP, Kumar PCK, Murray HW (1998). Rapid accurate field diagnosis of Indian visceral leishmaniasis. Lancet.

[8] Mabey D, Peeling RW, Ustianowski A, Perkins MD (2004). Diagnostics for the developing world. Nat Rev Microbiol.

[9] Joseph L, Gyorkos TW, Coupal L (1995). Bayesian estimation of disease prevalence and the parameters of diagnostic tests in the absence of a gold standard. Am J Epidemiol.

[10] Speybroeck N, Praet N, Claes F, van Hong N, Torres K, Mao S (2011). True versus apparent malaria infection prevalence: the contribution of a Bayesian approach. PLoS One.

[11] Speybroeck N, Devleesschauwer B, Joseph L, Berkvens D (2013). Misclassification errors in prevalence estimation: Bayesian handling with care. Int J Public Health.

[12] Neuhaus JM (1999). Bias and efficiency loss due to misclassified responses in binary regression. Biometrika.

[13] Duffy SW, Warwick J, Williams ARW, Keshavarz H, Kaffashian F, Rohan TE (2004). A simple model for potential use with a misclassified binary outcome in epidemiology. J Epidemiol Commun Health.

[14] Chen Q, Galfalvy H, Duan N (2013). Effects of disease misclassification on exposure-disease association. Am J Public Health.

[15] Magder LS, Hughes JP (1997). Logistic regression when the outcome is measured with uncertainty. Am J Epidemiol.

[16] Lyles RH, Tang L, Superak HM, King CC, Celentano DD, Lo Y (2011). Validation data-based adjustments for outcome misclassification in logistic regression: an illustration. Epidemiology.

[17] Fox MP, Lash TL, Greenland S (2005). A method to automate probabilistic sensitivity analyses of misclassified binary variables. Int J Epidemiol.

[18] McInturff P, Johnson WO, Cowling D, Gardner IA (2004). Modelling risk when binary outcomes are subject to error. Stat Med.

[19] Valle D, Clark J (2013). Improving the modeling of disease data from the government surveillance system: a case study on malaria in the Brazilian Amazon. PLoS Comput Biol.

[20] Stamey JD, Young DM, Seaman JW (2008). A Bayesian approach to adjust for diagnostic misclassification between two mortality causes in Poisson regression. Stat Med.

[21] O’Meara WP, Barcus M, Wongsrichanalai C, Muth S, Maguire JD, Jordan RG (2006). Reader technique as a source of variability in determining malaria parasite density by microscopy. Malar J.

[22] Valle D, Clark J, Zhao K (2011). Enhanced understanding of infectious diseases by fusing multiple datasets: a case study on malaria in the Western Brazilian Amazon region. PLoS One.

[23] Silva-Nunes MD, Codeco CT, Malafronte RS, da Silva NS, Juncansen C, Muniz PT (2008). Malaria on the Amazonian frontier: transmission dynamics, risk factors, spatial distribution, and prospects for control. Am J Trop Med Hyg..

[24] Ladeia-Andrade S, Ferreira MU, de Carvalho ME, Curado I, Coura JR (2009). Age-dependent acquisition of protective immunity to malaria in riverine populations of the Amazon Basin of Brazil. Am J Trop Med Hyg.

[25] Alves FP, Durlacher RR, Menezes MJ, Krieger H, da Silva LHP, Camargo EP (2002). High prevalence of asymptomatic *Plasmodium vivax* and *Plasmodium falciparum* infections in native Amazonian populations. Am J Trop Med Hyg.

[26] Mtove G, Nadjm B, Amos B, Hendriksen ICE, Muro F, Reyburn H (2011). Use of an HRP2-based rapid diagnostic test to guide treatment of children admitted to hospital in a malaria-endemic area of north-east Tanzania. Trop Med Int Health.

[27] Onchiri FM, Pavlinac PB, Singa BO, Naulikha JM, Odundo EA, Farguhar C (2015). Frequency and correlates of malaria over-treatment in areas of differing malaria transmission: a cross-sectional study in rural Western Kenya. Malar J.

[28] van Genderen PJJ, van der Meer IM, Consten J, Petit PLC, van Gool T, Overbosch D (2005). Evaluation of plasma lactate as a parameter for disease severity on admission in travelers with *Plasmodium falciparum* malaria. J Travel Med.

[29] Alonzo TA, Pepe MS, Lumley T (2003). Estimating disease prevalence in two-phase studies. Biostatistics.

[30] Halloran ME, Longini IM (2001). Using validation sets for outcomes and exposure to infection in vaccine field studies. Am J Epidemiol.

[31] Black MA, Craig BA (2002). Estimating disease prevalence in the absence of a gold standard. Stat Med.

[32] Brenner H (1996). Correcting for exposure misclassification using an alloyed gold standard. Epidemiology.

[33] Tang L, Lyles RH, King CC, Celentano DD, Lo Y (2015). Binary regression with differentially misclassified response and exposure variables. Stat Med.

[34] Tang L, Lyles RH, King CC, Hogan JW, Lo Y (2015). Regression analysis for differentially misclassified correlated binary outcomes. J R Stat Soc Ser C Appl Stat.

[35] Richardson BB, Hughes JP (2000). Product limit estimation for infectious disease data when the diagnostic test for the outcome is measured with uncertainty. Biostatistics.

