# Supplementary material for: Bias in logistic regression due to imperfect diagnostic test results and practical correction approaches
Source: Malar J. 2015 Nov 4;14:434. doi: 10.1186/s12936-015-0966-y (PMC4634725; doi:10.1186/s12936-015-0966-y)
Supplement: Supplementary file 1 — 10.1186/s12936-015-0966-y Code for the Bayesian models used in the main manuscript. [file 12936_2015_966_MOESM1_ESM.pdf]

S1 APPENDIX. CODE FOR THE BAYESIAN MODELS USED IN THE MAIN MANUSCRIPT

We rely on JAGS [1] to fit these models and invoke this software from within R [2]. To enable readers to implement our models, we provide below the corresponding R code and the associated JAGS file (extension ".bug" file). We also provide simulated data to be used for each of these models. These data are stored in the files 'fake data mod1.csv', 'fake data mod2.csv', and 'fake data mod3.csv'.

1) **Bayesian model 1:**

R code used to invoke JAGS, assuming $\widehat{SN} = 0.7$ and $\widehat{SP} = 0.95$:

```
rm(list=ls(all=TRUE))
library('rjags')

#import data
setwd('U:/detection/Bmodels/mod1')
data1=read.csv('fake data mod1.csv',as.is=T)
t1=data1$t1       #binary outcome
x1=data1$x1       #covariate 1
x2=data1$x2       #covariate 2
x3=data1$x3       #covariate 3
x4=data1$x4       #covariate 4
N=nrow(data1)     #number of observations
SN=0.7
SP=0.95

#to get initial values for regression parameters
zz=glm(t1~.,data=data1,family='binomial')
betas=zz$coef

#sets up model object
jags=jags.model('bm1.bug',data=list('t1'=t1,'x1'=x1,'x2'=x2,'x3'=x3,'x4'=x4,'N'=N,'SP'=SP,'SN'=SN),n.chains=4,n.adapt=500,inits=l
ist('betas'=betas))

#burn-in period
update(jags,1000)

#draw 4,000 samples from the sampler
res=jags.samples(jags,c('betas'),4000)
```

JAGS file ('bm1.bug') containing the model specification:

```
model{
	for (i in 1:N){
		#calculation of probability of disease p1
		tmp[i]<-exp(betas[1]+betas[2]*x1[i]+betas[3]*x2[i]+betas[4]*x3[i]+betas[5]*x4[i])
		p1[i]<-tmp[i]/(1+tmp[i])
		#modeling disease status d1
		d1[i]~dbern(p1[i])

		#probability of getting a positive diagnostic test result p2
		p2[i]<-ifelse(d1[i]==1,SN,1-SP)
		#modeling of diagnostic test result t1
		t1[i]~dbern(p2[i])
	}

	#priors
	for (i in 1:5){
		betas[i] ~ dnorm(0,1/10)
	}
}
```

2) <u>Bayesian model 2</u>

R code used to invoke JAGS, assuming $N_+ = 200, T_+ = 140, N_- = 200$, and $T_- = 190$:

```
rm(list=ls(all=TRUE))
library('rjags')

#import data
setwd('U:/detection/Bmodels/mod2')
data1=read.csv('fake data mod2.csv',as.is=T)
t1=data1$t1       #binary outcome
x1=data1$x1       #covariate 1
x2=data1$x2       #covariate 2
x3=data1$x3       #covariate 3
x4=data1$x4       #covariate 4
N=nrow(data1)     #number of observations
nplus=200         #number of individuals detected to be infected by gold standard method
tplus=nplus*0.7   #number of individuals detected to be infected by employed method (subset of nplus)
nminus=200        #number of individuals not detected to be infected by the gold standard method
tminus=nminus*0.95 #number of individuals not detected to be infected by the employed method (subset of nminus)

#to get initial values for regression parameters
zz=glm(t1~.,data=data1,family='binomial')
betas=zz$coef

setwd('U:/detection/Bmodels/mod2')
#sets up model object
jags=jags.model('bm2.bug',data=list('t1'=t1,'x1'=x1,'x2'=x2,'x3'=x3,'x4'=x4,
                            'N'=N,'nplus'=nplus,'nminus'=nminus,
                            'tplus'=tplus,'tminus'=tminus),n.chains=4,n.adapt=500,inits=list('betas'=betas))

#burn-in period
update(jags,1000)

#draw 4,000 samples from the sampler
res=jags.samples(jags,c('betas'),4000)
```

JAGS file (‘bm2.bug’) containing the model specification:

```
model{
	for (i in 1:N){
		#calculation of probability of disease p1
		tmp[i]<-exp(betas[1]+betas[2]*x1[i]+betas[3]*x2[i]+betas[4]*x3[i]+betas[5]*x4[i])
		p1[i]<-tmp[i]/(1+tmp[i])
		#modeling disease status d1
		d1[i]~dbern(p1[i])

		#probability of getting a positive diagnostic test result p2
		p2[i]<-ifelse(d1[i]==1,SN,1-SP)
		#modeling of diagnostic test result t1
		t1[i]~dbern(p2[i])
	}

	#uninformative priors for regression parameters
	for (i in 1:5){
		betas[i] ~ dnorm(0,1/10)
	}

	#informative priors for sensitivity (SN) and specificity (SP)
	SN ~ dbeta(tplus+1,nplus-tplus+1)
	SP ~ dbeta(tminus+1,nminus-tminus+1)
}
```

3) Bayesian model 3

The R code to invoke JAGS assumes that the first NG individuals are part of the internal validation sample and thus these individuals have results from the regular diagnostic method “t1” and the gold standard method “g1”:

```
rm(list=ls(all=TRUE))
library('rjags')

#import data
setwd('U:/detection/Bmodels/mod3')
data1=read.csv('fake data mod3.csv',as.is=T)
t1=data1$t1       #binary outcome from regular diagnostic method
g1=data1$g1       #binary outcome from gold standard diagnostic method
x1=data1$X1       #covariate 1
x2=data1$X2       #covariate 2
x3=data1$X3       #covariate 3
x4=data1$X4       #covariate 4
N=nrow(data1)     #number of observations
NG=sum(!is.na(g1)) #number of observations with gold standard diagnostic test results

#to get initial values for regression parameters
zz=glm(g1~.,data=data1[,c('g1','X1','X2','X3','X4')],family='binomial')
betas=zz$coef

#to get initial values for disease status
d1=g1
cond=is.na(d1)
d1[cond]=t1[cond]
d1[1:NG]=g1[1:NG]

#sets up model object
jags=jags.model('bm3.bug',data=
        list('t1'=t1,'g1'=g1,'x1'=x1,'x2'=x2,'x3'=x3,'x4'=x4,'N'=N,'NG'=NG),
        n.chains=4,n.adapt=500,inits=list('alpha'=rep(0,2),'omega'=rep(0,2),'betas'=betas,'d1'=d1))

#burn-in period
update(jags,1000)

#draw 4,000 samples from the sampler
results=jags.samples(jags,c('alpha','omega','betas'),4000)
```

Corresponding JAGS file (‘bm3.bug’), containing the full model specification:

```
model{
	for (i in 1:N){
		#calculation of probability of disease p1
		tmp[i]<-exp(betas[1]+betas[2]*x1[i]+betas[3]*x2[i]+betas[4]*x3[i]+betas[5]*x4[i])
		p1[i]<-tmp[i]/(1+tmp[i])
		#modeling disease status d1
		d1[i]~dbern(p1[i])

		#calculation of sensitivity SN, assuming it is only affected by x1
		tmp1[i]<- exp(alpha[1]+ alpha[2]*x1[i])
		SN[i]<-tmp1[i]/(1+tmp1[i])

		#calculation of specificity SP, assuming it is only affected by x1
		tmp2[i]<- exp(omega[1]+omega[2]*x1[i])
		SP[i]<- tmp2[i]/(1+tmp2[i])

		#probability of getting a positive diagnostic test result p2
		p2[i]<-ifelse(d1[i]==1,SN[i],1-SP[i])
		#modeling of diagnostic test result t1
		t1[i]~dbern(p2[i])
	}

	#to ensure that disease status is equal to gold standard results (d1=g1) for internal validation sample
	for (i in 1:NG){
		p3[i]<-ifelse(d1[i]==1,1,0)
		g1[i]~dbern(p3[i])
	}
	#uninformative priors for regression parameters
	for (i in 1:5){
		betas[i] ~ dnorm(0,1/10)
	}
	for (i in 1:2){
		alpha[i] ~ dnorm(0,1/10)
		omega[i] ~ dnorm(0,1/10)
	}
}
```

References

1. Plummer M (2013) Package 'rjags'. The comprehensive R archive network.
2. R Core Team (2013) R: A language and environment for statistical computing.: R Foundation for Statistical Computing, Vienna, Austria. http://www.R-project.org/.
